# Characterization of ADME genes variation in Roma and 20 populations worldwide

**DOI:** 10.1371/journal.pone.0207671

**Published:** 2018-11-19

**Authors:** Tatjana Škarić-Jurić, Željka Tomas, Matea Zajc Petranović, Nada Božina, Nina Smolej Narančić, Branka Janićijević, Marijana Peričić Salihović

**Affiliations:** 1 Institute for Anthropological Research, Zagreb, Croatia; 2 Department for Pharmacogenomics and Therapy Individualization, University Hospital Center Zagreb, Department of Pharmacology, University of Zagreb School of Medicine, Zagreb, Croatia; National Cheng Kung University, TAIWAN

## Abstract

The products of the polymorphic ADME genes are involved in Absorption, Distribution, Metabolism, and Excretion of drugs. The pharmacogenetic data have been studied extensively due to their clinical importance in the appropriate drug prescription, but such data from the isolated populations are rather scarce. We analyzed the distribution of 95 polymorphisms in 31 core ADME genes in 20 populations worldwide and in newly genotyped samples from the Roma (Gypsy) population living in Croatia. Global distribution of ADME core gene loci differentiated three major clusters; (1) African, (2) East Asian, and (3) joint European, South Asian and South American cluster. The *SLCO1B3* (rs4149117) and *CYP3A4* (rs2242480) genes differentiated at the highest level the African group of populations, while *NAT2* gene loci (rs1208, rs1801280, and rs1799929) and *VKORC1* (rs9923231) differentiated East Asian populations. The *VKORC1* rs9923231 was among the investigated loci the one with the largest global minor allele frequency (MAF) range; its MAF ranged from 0.027 in Nigeria to 0.924 in Han Chinese. The distribution of the investigated gene loci positions Roma population within the joined European and South Asian clusters, suggesting that their ADME gene pool is a combination of ancestral (Indian) and more recent (European) surrounding, as it was already implied by other genetic markers. However, when compared to the populations worldwide, the Croatian Roma have extreme MAF values in 10 out of the 95 investigated ADME core gene loci. Among loci which have extraordinary MAFs in Roma population two have strong proof of clinical importance: rs1799853 (*CYP2C9*) for warfarin dosage, and rs12248560 (*CYP2C19*) for clopidogrel dosage, efficacy and toxicity. This finding confirms the importance of taking the Roma as well as the other isolated populations`genetic profiles into account in pharmaco-therapeutic practice.

## Introduction

Medication efficacy and adverse drug reactions are associated with specific genes’ variants [1] that are related to the Absorption, Distribution, Metabolism and Elimination of drugs (ADME). Their polymorphic nature is the basis for the individual response to drug treatment, together with a number of factors such as sex, age, weight, concomitant medication, health status, comorbidity level etc. [2]. The ADME genes' variation is markedly related to ethnicity and shows distinct geographic patterns [2, 3].

Numerous studies have been published on ADME-related genes' polymorphisms and their clinical importance. Some of them used large panels of genes and/or big samples from large population groups [1, 4–10], while others provided data for specific variants of interest and/or smaller samples from diverse geographic or clinical populations [11–14]. Currently, only sparse data are available on the prevalence of these gene variants in Roma (Gypsy) population [15–17]. Lacking large-scale pharmacogenomics information in this population presents a bottleneck for their healthcare improvement.

Although today’s state-of-art methodology in personalized medicine is individual genotyping prior to medications, unfortunately this approach has not been routinely applied at points of care and usually follows after an adverse drug effect. Therefore, population ADME genes profiling is useful for clinical practice especially in cases when the population consists of different ancestry groups.

It has been estimated that about 15 million of Roma people live worldwide today from whom 10 million reside in Europe. About 40 thousand Roma live in Croatia [18]. However, their numbers are probably significantly underestimated due to ethnomimicry characteristically present in this population in addition to the avoidance of contacts with state officials, leaving many members of this population unregistered in census data.

Anthropologically, the Roma are the transnational minority population marked by common Indian ancestry. Various social and economic pressures caused gradual population fragmentation and formation of a complex network of numerous and often endogamous subgroups with specific languages (dialects), religions, and socio-cultural characteristics [19, 20]. Lasting isolation preserved their founding gene pool with different characteristics compared to the surrounding majority populations [21].

Their pronounced genetic differences from the majority Croatian population and traces of their ancestral origins have been detected in mitochondrial DNA [22], Y chromosome markers [23, 24] and various autosomal common [25] and rare disease loci [26]. The recent study of ADME gene's CYP2B6 polymorphisms proved the distinctive position of the Croatian Roma in the world's populational variability [27] and indicated the need for a systematic investigation of the most important pharmacogenes' variants in the Roma.

Since isolated populations usually have a unique genetic profile it is important to determine their ADME genes pattern in the context of broader global diversity [28, 29]. Therefore, in this study, we (1) present the allele frequencies for 95 polymorphisms in 31 core ADME-related genes for 20 worldwide populations as well as for the Roma population living in Croatia; (2) identify and describe the set of markers that mostly contribute to the separation of major population groups and, thus, give rise to specific geographic patterns of core ADME gene loci; and (3) elucidate the position of the Roma in the global ADME genetic landscape.

## Results

The minor allele frequencies (MAFs) and sample sizes as well as the references in addition to the 1000 Genomes for all 95 SNPs used in the this study are presented in the S1 Table. MAF always refers to the global minor allele as indicated in 1000 Genomes’ database.

**Fig 1:**
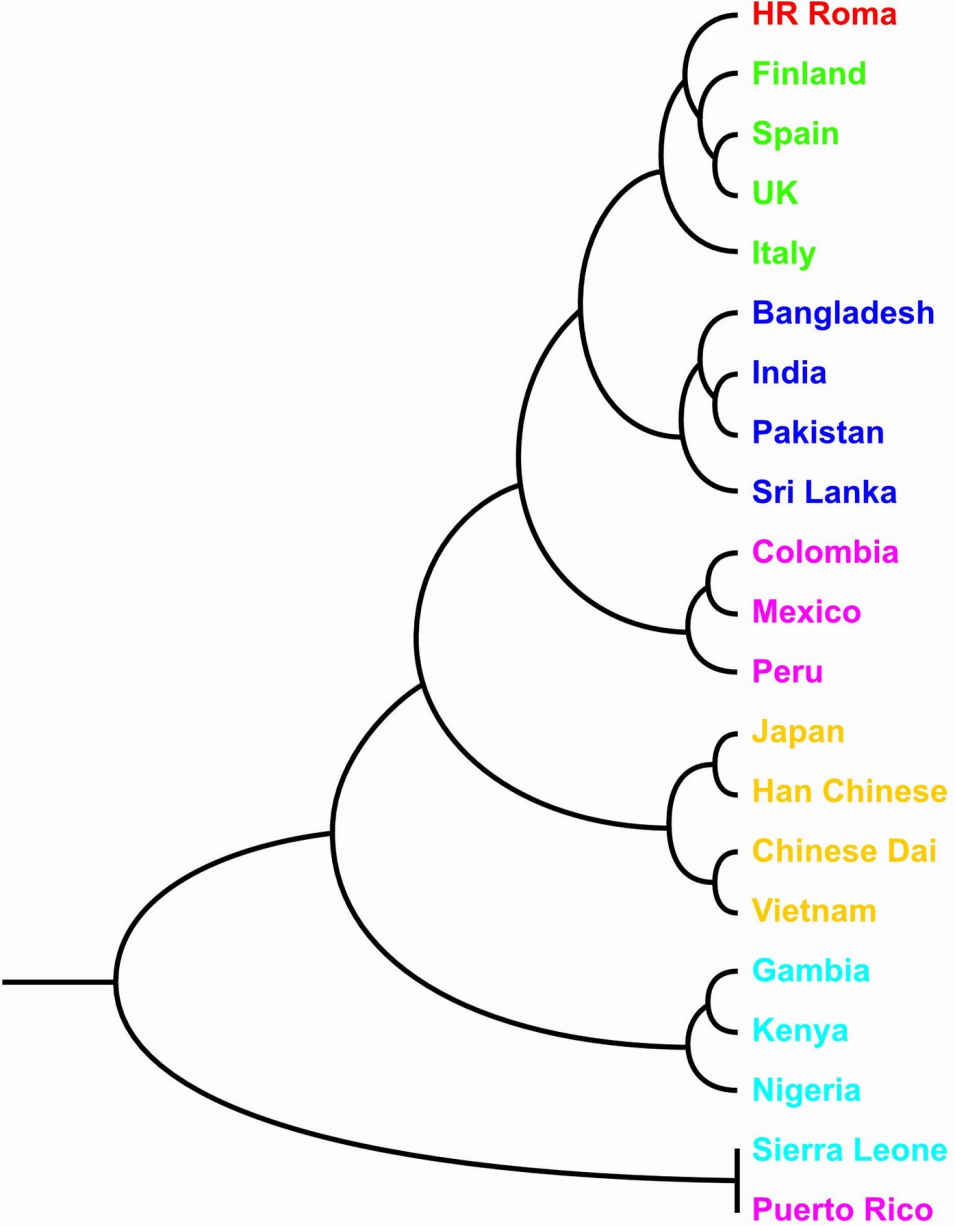
UPGMA dendrogram from Nei’s genetic distance matrix based on the data on minor allele frequencies for 95 ADME core genes’ loci in Croatian Roma and 20 populations worldwide.

Genetic distance analyses were carried out to quantify genetic differentiation across 21 populations in this study and the dendrogram is reported in Fig 1. As expected, there is a clear separation of clusters that correspond to the continental regions their member populations belong. The European and the South Asian populations cluster closely together. They are joined successively by the American, East Asian and African clusters. Finally, closely joined Sierra Leone and Puerto Rico populations cluster as a distinct subgroup further away from the rest of the populations.

**Fig 2:**
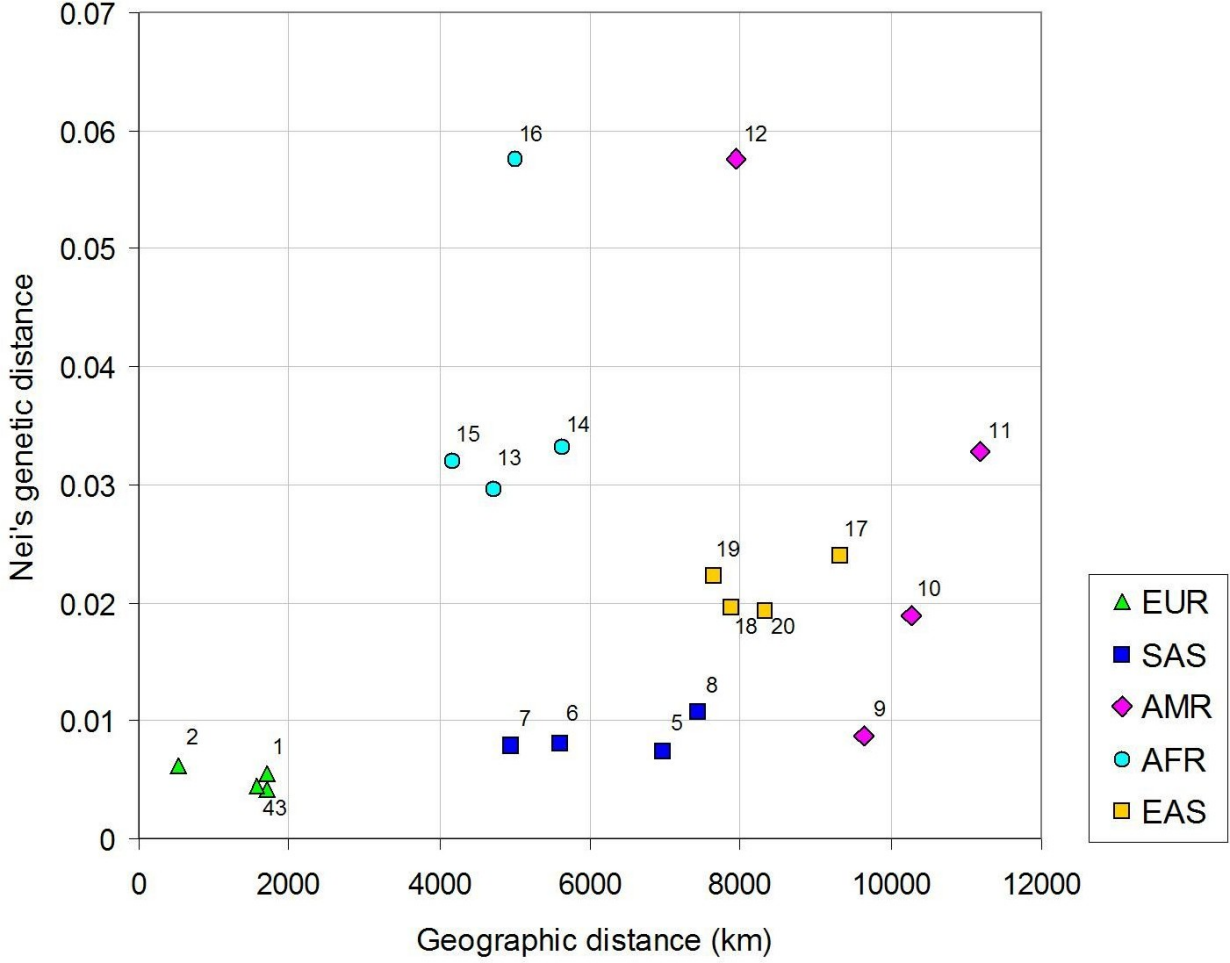
Genetic distances between the Croatian Roma and 20 populations worldwide in relation to their geographic distances. Legend: 1 = Finland; 2 = Italy; 3 = Spain; 4 = UK; 5 = Bangladesh; 6 = India; 7 = Pakistan; 8 = Sri Lanka; 9 = Colombia; 10 = Mexico; 11 = Peru; 12 = Puerto Rico; 13 = Gambia; 14 = Kenya; 15 = Nigeria; 16 = Sierra Leone; 17 = Japan; 18 = China–Dai; 19 = China–Han; 20 = Vietnam.

The relationship between genetic variation and geographic distance was analyzed using correlation between matrices of genetic and geographic distances. The correlation is positive and significant (Pearson`s r = 0.300, p≤0.006 after 1,000 permutations) indicating isolation by spatial distance at the global scale. Focusing on the Croatian Roma population, their genetic distances from the other 20 populations in this study were plotted against the geographic distances (Fig 2). Roma cluster well within the European populations, and are relatively close to the South Asian populations genetically despite their spatial distance of 5,000–7,500 km. Larger genetic distances exist between the Roma and spatially more distant East Asian as well as fairly dispersed American populations. Roma genetically differ the most from the African populations that are on the average closer to them geographically, confirming the genetic distinctiveness of the African region.

The Principal Component Analysis (PCA) was performed using MAF data for 95 ADME core genes’ loci for 21 populations from various part of the world, including Croatian Roma. PCA revealed six PCs (76.4% of the total variance explained) reflecting the genetic relationships among the populations showing a pattern that is very similar to that obtained by genetic distance dendrogram. PC1 (28.4% of the total variance) separates four African countries from the rest of the world while PC2 (20.9% of the total variance) separates four East Asian countries. PC3 (accounting for 10.8% of the total genetic variance) separates South Asians from the remaining European-American cluster with the Croatian Roma being intermediate to Europeans and South Asians. The PC4 axis (accounting for 8.5% of the total variance) separates Americans from the European-Roma group and places the Roma population at the top of the positive pole of the axis (while negative pole is represented by Peru). PC5 (explains 4.3% of the total variance) differentiates European countries (placing at the opposite poles Finland and UK) while PC6 (accounting for 3.5% of the total variance) differentiates East Asian populations (opposing Han from Dai Chinese populations).

In order to elucidate the most characteristic continental single nucleotide polymorphisms (SNPs) among the core ADME genes’ loci, we performed the gene-oriented Principal Component Analysis (gPCA). The gPCA revealed three significant components: gPC1 (explaining 77% of the total variance) was defined by the global range in MAF values (i.e. it contains not population-specific but locus-specific information). However, gPC2 (9.3%) and gPC3 (6.1% of the total variance) were population-specific.

**Fig 3:**
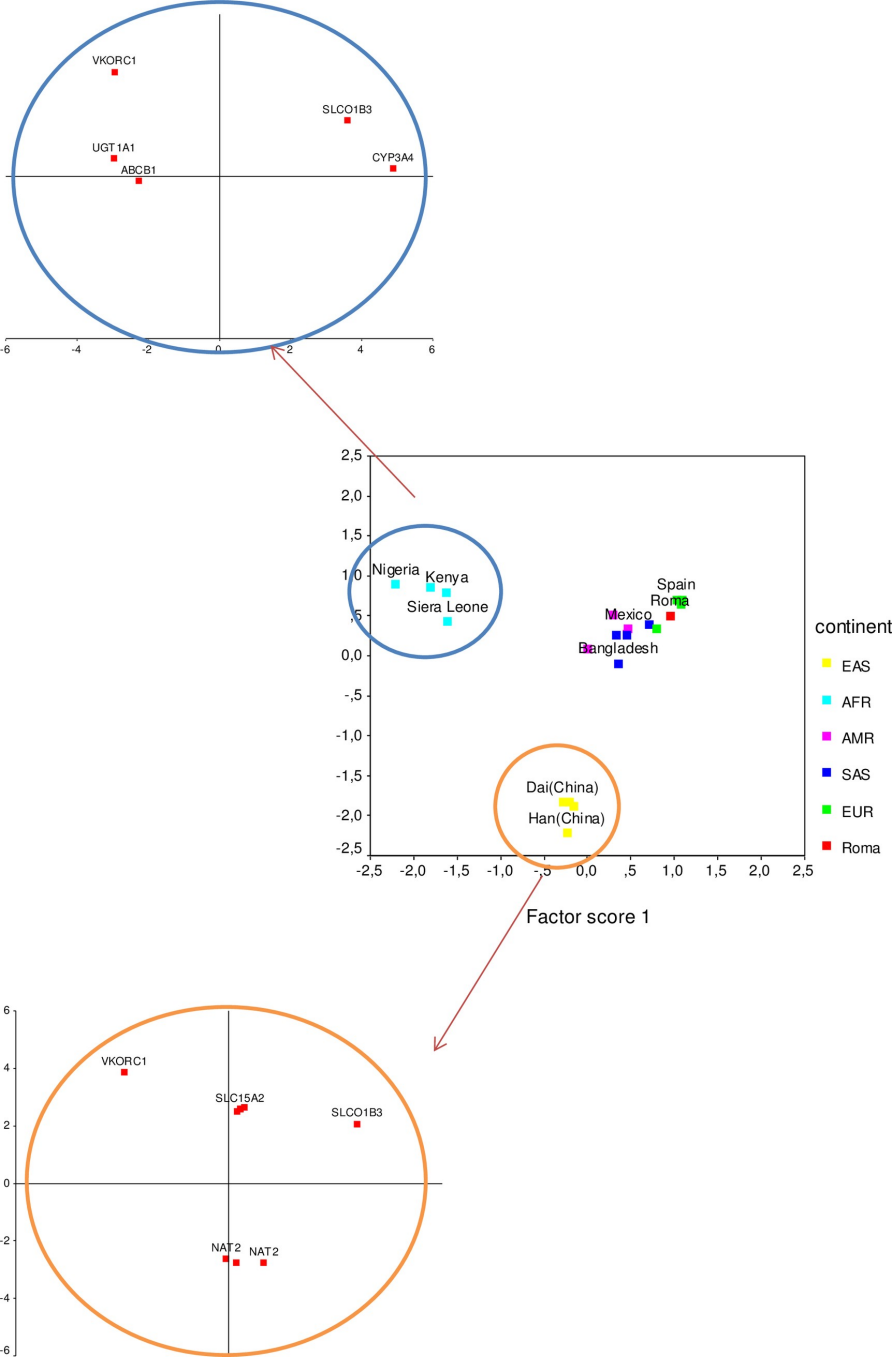
Principal component analyses (PCA) using the allele frequencies of the ADME core genes’ loci. Scatterplot illustrates the grouping of 21 populations by the first two principal components of the population-based PCA and shows the loci with the largest factor score values as revealed by the gene-based PCA (gPC2 and gPC3).

Combining population- and gene-oriented approaches, Fig 3 shows scatterplot of the first two principal components from the PCA together with the presentation of the loci with the highest factor scores in the gPCA. The first two principal components (PCs) of the PCA, accounting for 49.3% of the total genetic variance, clearly separate three clusters that reflect the major genetic relationships among the populations: the African (AFR), the East Asian (EAS) and the joint South Asian, European and American (SAS, EUR and AMR, respectively). The AFR and EAS groups are related with the ADME genes that have the highest factor scores at gPC2 and gPC3, respectively.

As shown in Fig 3, the most distinctive African loci are rs4149117 (*SLCO1B3*) and rs2242480 (*CYP3A4*) that have the highest factor scores at the positive pole and (with smaller factor score values) rs4124874 (*UGT1A1*), rs9923231 (*VKORC1*), and rs1128503 (*ABCB1*) at the negative pole. In the combination with the results (factor loadings) from the population-oriented PCA (data not here presented), we consider rs4149117 (*SLCO1B3*) and rs2242480 (*CYP3A4*) as the most distinctive African loci.

The East Asian (EAS) gPC3 has 6 loci on the positive pole (factor scores higher than 2): rs9923231 (*VKORC1*), rs1143671, rs2257212, rs1143672, rs2293616 (all four placed within *SLC15A2*), and rs4149117 (*SLCO1B3*). At the negative pole of gPC3 there are three SNPs (rs1208, rs1801280, and rs1799929) all of them placed within *NAT2* gene. In the combination with the results of the population-based PCA (data not here presented), those results indicate that distinctive SNPs for the East Asians are the three *NAT2* gene SNPs and a *VKORC1* (rs9923231) SNP.

**Fig 4:**
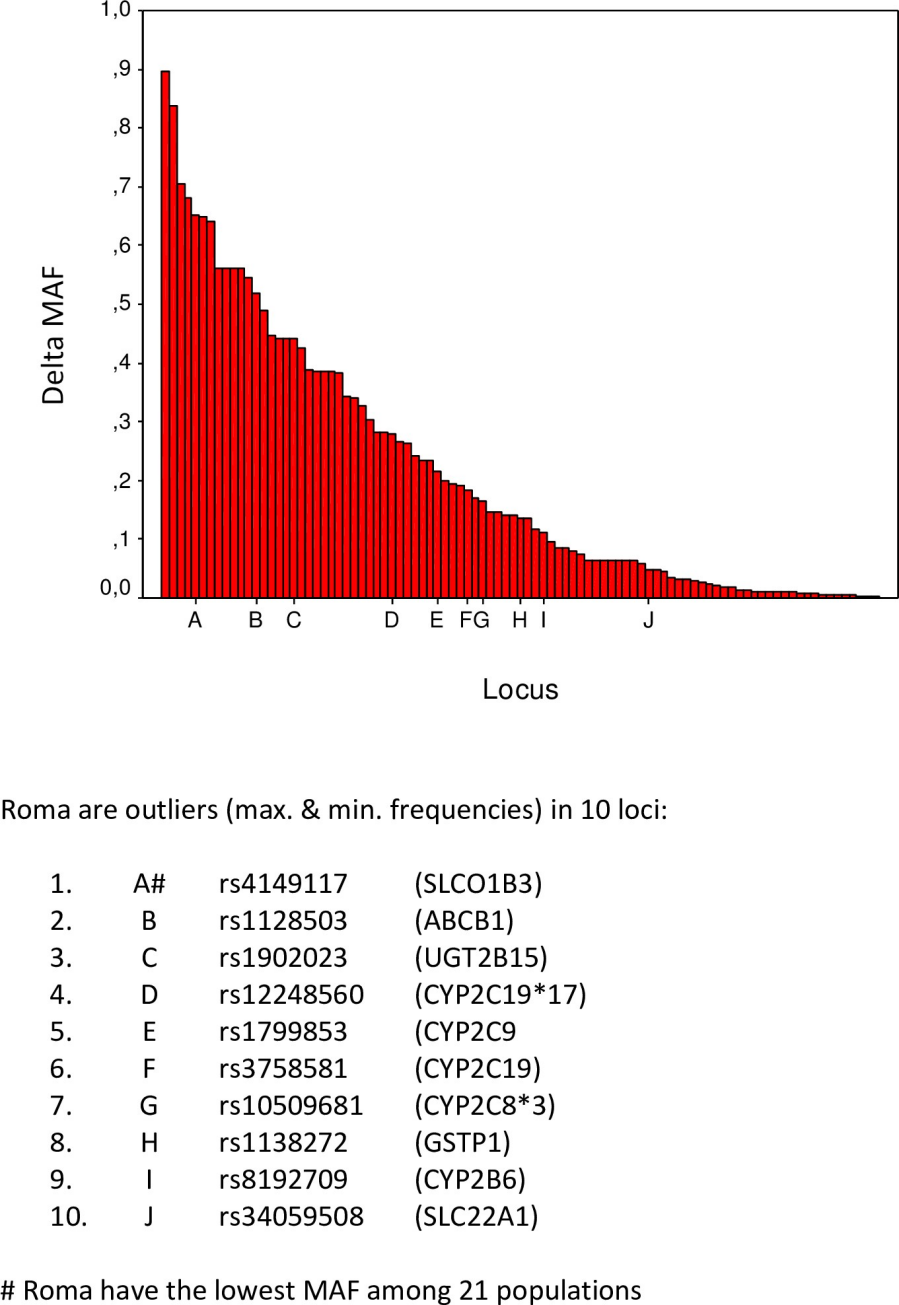
The maximal global differences in minor allele frequencies (delta) for the selected 95 ADME core genes’ loci in decreasing order.

The largest global MAF differences (delta) between maximal and minimal MAF values for 95 SNPs are presented graphically in decreasing order (Fig 4) and the populations with extreme MAF values as well as the exact delta values are given in the S2 Table. The three SNPs characterized with the largest global diversity are: rs9923231 in *VKORC1* gene (range: 0.924 in China Han to the 0.027 in Nigeria, delta = 0.897), rs2242480 in *CYP3A4* gene (range: 0.909 in Kenya to the 0.071 in UK; delta = 0.838) and rs1048943 in CYP1A1*2C gene (range: 0.706 in Peru to the 0.000 in Gambia, Kenya, Nigeria, and Sierra Leone; delta = 0.706).

Focusing on the Croatian Roma, they have the extreme MAF values for 10 SNPs (Fig 4). Their highest MAFs among 21 world’s populations have been found for rs1128503 (*ABCB1*), rs1902023 (*UGT2B15*), rs12248560 (CYP2C19*17), rs1799853 (*CYP2C9*), rs3758581 (*CYP2C19*), rs10509681 (CYP2C8*3), rs1138272 (*GSTP1*), rs8192709 (*CYP2B6*), and rs34059508 (*SLC22A1*) (and next to the highest MAF for rs28371725 (*CYP2D6*)). On the other side, Roma have the lowest MAF for rs4149117 (*SLCO1B3*) (and next to the lowest MAF value for the rs28399433 (*CYP2A6*)).

## Discussion

The findings of numerous studies suggest that population differences in ADME genes show marked geographic and ethnic variation. However, most of the studies investigating these variations generally lack pharmacogenetic data on isolated populations. The same is true for the Roma, one of the worlds’ largest transnational minority populations. Roma are an example of isolated population with specific migrational history whose gene pool is highly influenced by the genetic drift due to unique social and cultural features.

Therefore, in this paper we investigated the position that the Croatian Roma take within the world-wide variation in 95 ADME core genes’ loci. The ADME genes’ MAF information from twenty 1000 Genomes’ populations is here enriched by the data found through systematic literature search.

The results of this study confirm the previous findings that distinguish three world’s populational clusters: African, East Asian and joint European, South Asian and Native American [30]. Additionally, this study identifies six ADME genes’ SNP loci that most prominently distinguish continental groups which extends our knowledge about ADME global variation. They are: *VKORC1* (rs9923231), *SLCO1B3* (rs4149117), *CYP3A4* (rs2242480), and *NAT2* (rs1208, rs1801280, and rs1799929).

Among all the studied loci, locus rs9923231 in VKORC1 gene takes a special position. It is the one characterized by the largest MAF differences among the populations worldwide (delta = 0.897) and is among few SNPs that define both African and East Asian continental clusters. Those findings are even more important having in mind that among the ADME genes VKORC1 is the one with the most vital implications for medical decisions considering appropriate pharmacotherapy.

Striking differences in worldwide allele frequency distribution noticed in rs9923231 is mostly explained as a result of selection [31]. The minor allele frequency of 0.4614 in Roma is comparable to European populations and it is higher than in their ancestral south Asian populations. *VKORC1* gene encodes vitamin K 2,3-epoxide reductase complex 1, which is responsible for the conversion of vitamin K-epoxide to vitamin K [32]. Numerous studies suggest that *VKORC1* genotype seems to be the most important predictor of adequate warfarin dose [33, 34]. The rs9923231 has proved clinical importance concerning dosage and toxicity of warfarin, acenocoumarol and phenprocoumon.

Another locus, which shows large differences in allele frequencies among the investigated populations, is rs2242480, the intron variant in *CYP3A4* gene. This locus exhibits significant difference between Africans and non-Africans [35] which is evident in our gPC plot as well. Lakiotaki et al. [36] identified this variant to belong to the 10 most different ones in worldwide populations. Unlike the previously mentioned locus in VKORC1, the frequency of globally minor allele of rs2242480 in the investigated Roma population (0.3012) is higher than in European populations and corresponds to the frequency range of South Asian populations. CYP3A4 is responsible for the metabolism of approximately 50–60% of clinical drugs used today, including acetaminophen, codeine, cyclosporine A, diazepam, and erythromycin. It is also important for the metabolism of steroid hormones [33, 34]. Although there is no clear evidence of an association, the rs2242480 is suspected to be connected with methadone toxicity [37], clopidogrel efficacy [38] and pharmacokinetics of tacrolimus [39] carbamazepine [40].

Locus rs4149117 in *SLCO1B3* gene also separates African cluster from the other populations. The G allele of the rs4149771 locus was found even two times more frequently in non-Africans (Europeans, Caucasian Americans, and Asians) than in Africans, in approximately 80% vs. 40% of subjects [41, 42]. Roma population from Croatia has the lowest minor allele frequency in Europe. *SLCO1B3* gene encodes for solute carrier organic anion transporter family member 1B3 normally expressed in the liver and involved in transporter functions to uptake large, non-polar drugs and hormones. The rs4149771, like the previous one, is suspected to be connected with carboplatin and paclitaxel toxicity [43] and with sunitinib efficacy [44].

NAT2, one of the most polymorphic ADME genes, encodes for a NAT2 protein, which is expressed mostly in the liver, small intestine and colon tissues as a typical xenobiotic metabolizing enzyme [45]. NAT2 gene variants differ among diverse populations and its genetic differentiation patterns are related to geography [46]. The minor alleles’ frequencies of three NAT2 loci (rs1208, rs1801280, and rs1799929) in Roma sample are within the range of European populations. Isoniazid, a first line drug in tuberculosis (TB) treatment is metabolized by the NAT2 enzyme. Genetic variations in NAT2 affect the therapeutic response to isoniazid and other drugs detoxified by this enzyme.

Among loci with the most pronounced population differences as revealed by MAF delta values is rs1048943 missense (Ile462Val) mutation within *CYP1A1* gene. CYP1A1 is a member of the CYP1 family and participates in the metabolism of numerous xenobiotics, as well as endogenous substrates [47]. CYP1A1 is a key enzyme in phase I metabolism of polycyclic aromatic hydrocarbons and in estrogen metabolism. This mutation defines haplotype CYP1A1*2C. The highest frequency of the mutated allele is noticed in the South American population from Peru while the absence of mutated allele is noticed in most of the African populations. Such distribution implies its introduction after the Out of Africa migrational event and therefore its current distribution probably results from genetic drift. Roma population has the frequency of minor allele within the European range which is substantially lower than in the ancestral South Asian populations.

Although there is evidence of selection for some of the above mentioned genes, the overall large allele variations between populations more often result from genetic drift, migrations and other demographic events [48].

As it can be seen from our results, ADME core loci separate African and East Asian clusters from other Euro-Asian and American populations. This pattern is also confirmed by clustering of genetic distances. The Roma population is positioned within the European cluster and is close to the South Asian populations. Such results suggest that Roma ADME gene pool is a combination of two main layers: ancestral (Indian) and more recent (European). This is also evident from the analyses of the uniparental genetic markers [22, 23]. Similarly, Melegh et al. [49] found that the Roma are located on a PCA cline between Europeans and South Asians, but closer to Europeans by analyzing genome-wide SNP loci.

Although Roma population is found to be a member of the closely related European and South Asian clusters, it has the extreme MAF values in 10 out of 95 analyzed SNPs. Significant genetic differentiation from general Europeans in SNPs in the *CYP2C* and *CYP2D* subfamily regions was also found in previous research of isolated populations in Europe (Roma, Basques, and Orcadians) [50]. Among the former analyses of ADME polymorphisms in Roma populations, Tomas et al. [27] particularly studied 3 SNP loci in the *CYP2B6* gene, Spikey et al. [51] studied 4 SNP loci in the MDR1 gene while Nagy et al. [15] analyzed 2 SNP loci in the *SLCO1B3* gene and all of them confirmed that the Roma differ considerably from geographically close majority populations, as well as from Indian populations in those particular loci. Such results are not surprising knowing Roma genetic history which is influenced by strict rules of group endogamy, reproductive isolation and specific mating practice and isolation over the past several centuries.

Our population genetics findings contribute to the knowledge of interpopulation differences in high-risk pharmacogenomics allele distribution. The Pharmacogenomics Knowledgebase (PharmGKB) is a source of clinically relevant information, including dosing guidelines, annotated drug labels, and potentially actionable gene–drug associations and genotype–phenotype relationships [52]. Several loci which have extraordinary MAFs in Roma population are listed in PharmGKB as loci with strong proof of clinical importance. Two of them have been clinically annotated as level 1A (strong evidence—included in the Clinical Pharmacogenetics Implementation Consortium–CPIC guidelines): rs1799853 (*CYP2C9*) for warfarin dosage, and rs12248560 (*CYP2C19*) for clopidogrel dosage, efficacy and toxicity, while the second locus has been also clinically annotated as level 2A (very important pharmacogene) for citalpram or escitalopram pharmacokinetics. Additionally, rs10509681 (*CYP2C8*) has been annotated as level 2A for rosiglitazone pharmacokinetics and rs1902023 (*UGT2B15*) as level 2B (moderate clinical evidence) for lorazepam or oxazepam (www.pharmagkb.org). The identification of high risk allele at loci whose genotypes have a direct influence on quality of drug intake in this population, shows the necessity of the assessment of unique genetic profile of Roma in order to achieve the most in the modulation of pharmacotherapy in this population.

Our data confirm that isolated populations take specific positions within the global ADME genetic landscape. This pinpoints that the pharmacogenetics guidelines of the well-defined majority populations cannot be used in pharmaco-therapeutic practice in population isolates, and confirms the necessity for defining their specific genetic profile.

## Material and methods

Biological material used in this study was collected in multiple field studies, which were part of the on-going multidisciplinary anthropological, molecular-genetic and epidemiological research of Roma populations in Croatia. The fieldwork was carried out in several regions of Croatia with the highest number of Roma minority inhabitants according to the census data [18]. The participants were volunteers and were informed about the goals, methods and expectations of the study with the help of linguistically and culturally competent and trained Roma volunteers. The study protocol was approved by the Scientific Board and the Ethical Committee of the Institute for Anthropological Research in Zagreb, Croatia.

Genotyping of 439 DNA samples was done using KASP method. The KASP genotyping assay is a form of competitive allele-specific PCR combined with homogeneous fluorescent SNP genotyping system, which determines the alleles at a specific locus within genomic DNA [53]. This technology has been widely used on plant species, while recently it has been successfully applied to human samples too [54, 55]. From the list of evidence-proved genetic biomarkers associated with metabolism of drugs, which is available at www.pharmaadme.org, 137 single nucleotide polymorphisms (SNPs) were selected for genotyping using the KASP and 127 of them were genotyped successfully. Allele and genotype frequencies were calculated by direct counting method.

The present investigation of genetic diversity was based on SNPs from the ADME core list which were genotyped in both the Croatian Roma and in 20 populations with different genetic ancestry from the 1000 Genomes Project Phase 3 list. This limitation and the finding that four ADME SNPs genotyped were monomorphic, led to further reduction of the total number of SNPs so in the end a total of 95 SNPs located in 31 ADME genes were used for the analyses.

The 20 populations from the 1000 Genomes project belong to the five large continental regions, and each region is represented by four populations: (1) European (EUR): Finland, Italy, Spain, UK, (2) South Asian (SAS): Bangladesh, India, Pakistan, Sri Lanka, (3) African (AFR): Gambia, Kenya, Nigeria, Sierra Leone, (4) Central and South American (AMR): Colombia, Mexico, Peru, Puerto Rico, and (5) Eastern Asian (EAS): Dai Chinese, Han Chinese, Japan, Vietnam (only China is represented by two distinct populations—Han and Dai—since Han is a majority population while Dai represent here non-Han China populations).

In our analyses, we enriched the 1000 Genomes’ data with those found in the publications citing any of the 95 investigated SNPs in the above mentioned populations. Selection criteria for using data from these publications, listed at the *e*!*Ensembl* browser for the each SNP, were: (1) clearly stated study geographical population and, where relevant, participants' ethnicity, (2) alleles frequencies and sample sizes, (3) samples come from the general population or control groups in case-control studies. These additional genotyping data enlarged the size of the 11 following 1000 Genomes populations: Italy, Spain, UK, India, Sri Lanka, Gambia, Kenya, Colombia, Mexico, Han Chinese and Japan. Allele frequencies for these populations were calculated by weighting samples for each population.

The genetic distance matrix, computed according to the method of Nei (1972), was subjected to hierarchic clustering routine using UPGMA (unweighted pair-group method using arithmetic averages) available in free software Phylip v3.697 (http://evolution.gs.washington.edu/phylip.html).

The Mantel test of correlation between genetic and geographic distances was performed using non-commercial software *IBD*: *Isolation by distance* v1.52 (available at http://www.bio.sdsu.edu/pub/andy/IBD.html). Geographic distances between the analyzed populations were calculated using two free online softwares: *iTouchMap* and *Movable Type Scripts*. *iTouchMap* calculates latitude and longitude of a point, and *Movable Type Scripts* calculated distance between latitude/longitude points (available at http://itouchmap.com/latlong.html and http://www.movable-type.co.uk/scripts/latlong.html).

Principal component analysis (PCA) is a multivariate method that systematically identifies underlying variables, or principal components (PCs), that best differentiate a set of data [56]. Two analyses were run using the MAF data of the 95 ADME core SNPs in 21 populations. First, PCA was performed to investigate the grouping of 21 populations using the known genetic data. The second analysis, the gene-oriented PCA (gPCA), was run to investigate the clustering of SNPs using the a priori defined 21 populations in order to detect loci defining the population clusters obtained in PCA. The number of PCs considered in each analysis was determined from the scree plot. This statistics was performed using the SPSS software package 17.0.

## Supporting information

S1 Table The minor allele frequency (MAF) weighted values and sample sizes for the selected 95 ADME core genes’ loci in Croatian Roma (present study) and in 20 populations worldwide. The references are provided in cases when data from the literature are used in addition to the 1000 Genomes data.
(XLSX)

S2 Table Populations with maximal and minimal minor allele frequencies (MAF) values for the selected 95 ADME core genes’ loci. The list is ordered by decreasing delta values (difference between maximal and minimal MAF).
(DOCX)
