# Supplementary material for: Characterization of ADME genes variation in Roma and 20 populations worldwide
Source: PLoS One. 2018 Nov 19;13(11):e0207671. doi: 10.1371/journal.pone.0207671 (PMC6242375; doi:10.1371/journal.pone.0207671)
Supplement: S2 Table — The list is ordered by decreasing delta values (difference between maximal and minimal MAF). (DOCX) [file pone.0207671.s002.docx]

Suppl. Table 2. Populations with maximal and minimal minor allele frequencies (MAF) values for the selected 95 ADME core genes’ loci. The list is ordered by decreasing delta values (difference between maximal and minimal MAF).

Legend: 1=Croatian Roma; 2=Finland; 3=Italy; 4=Spain; 5=UK; 6=Bangladesh; 7=India; 8=Pakistan; 9=Sri Lanka; 10=Gambia; 11=Kenya; 12=Nigeria; 13=Sierra Leone; 14=Colombia; 15=Mexico; 16=Peru; 17=Puerto Rico; 18=Japan; 19=China – Dai; 20=China –Han; 21=Vietnam

| Gene | rs | Max. MAF | Population with maximal MAF | Min. MAF | Population with Minimal MAF | Delta value |
| --- | --- | --- | --- | --- | --- | --- |
| VKORC1 | 9923231 | 0.9237 | 20 | 0.0266 | 12 | 0.8971 |
| CYP3A4 | 2242480 | 0.9090 | 11 | 0.0710 | 5 | 0.8380 |
| CYP1A1*2C | 1048943 | 0.7060 | 16 | 0.0000 | 10, 13 | 0.7060 |
| CYP1A2 | 2069514 | 0.6820 | 16 | 0.0023 | 1 | 0.6797 |
| SLCO1B3 | 4149117 | 0.7220 | 11 | 0.0701 | 1 (the next is 7) | 0.6519 |
| ABCC2 | 717620 | 0.6542 | 3 | 0.0060 | 13 | 0.6482 |
| UGT1A1 | 4124874 | 0.6880 | 18 | 0.0470 | 13 | 0.6410 |
| SLC15A2 | 1143671 | 0.7640 | 18 | 0.2030 | 15 | 0.5610 |
| SLC15A2 | 1143672 | 0.7640 | 18 | 0.2030 | 15 | 0.5610 |
| SLC15A2 | 2257212 | 0.7640 | 18 | 0.2030 | 15 | 0.5610 |
| SLC15A2 | 2293616 | 0.7640 | 18 | 0.2030 | 15 | 0.5610 |
| GSTP1 | 1695 | 0.6710 | 16 | 0.1270 | 18 | 0.5440 |
| ABCB1 | 1128503 | 0.6295 | 1, 18 | 0.1110 | 11 | 0.5185 |
| ABCB1 | 1045642 | 0.6100 | 6 | 0.1207 | 12 | 0.4893 |
| NAT2 | 1801280 | 0.4670 | 5 | 0.0190 | 18 | 0.4480 |
| DPYD | 1801265 | 0.5000 | 11 | 0.0580 | 18 | 0.4420 |
| NAT2 | 1208 | 0.4600 | 11 | 0.0190 | 18 | 0.4410 |
| UGT2B15 | 1902023 | 0.7138 | 1 | 0.2730 | 15 | 0.4408 |
| NAT2 | 1799929 | 0.4450 | 5 | 0.0190 | 18 | 0.4260 |
| CYP2B6 | 7260329 | 0.4690 | 20 | 0.0800 | 10 | 0.3890 |
| UGT2B7 | 7439366 | 0.5270 | 5 | 0.1410 | 16 | 0.3860 |
| SLC22A1 | 628031 | 0.4850 | 2 | 0.1000 | 16 | 0.3850 |
| CYP1A2 | 762551 | 0.5200 | 11 | 0.1350 | 16 | 0.3850 |
| CYP2D6 | 1135840 | 0.6060 | 16 | 0.2220 | 21 | 0.3840 |
| ABCG2 | 2231142 | 0.3430 | 21 | 0.0000 | 11, 12 | 0.3430 |
| NAT2 | 1799930 | 0.4220 | 9 | 0.0820 | 16 | 0.3400 |
| CYP2C19 | 4244285 | 0.3853 | 9 | 0.0590 | 16 | 0.3263 |
| ABCC2 | 3745274 | 0.4590 | 16 | 0.1570 | 2 | 0.3020 |
| NAT2 | 1041983 | 0.5400 | 21 | 0.2590 | 16 | 0.2810 |
| ABCC2 | 3740066 | 0.4327 | 4 | 0.1520 | 11 | 0.2807 |
| CYP2C19*17 | 12248560 | 0.2834 | 1 | 0.0050 | 18 | 0.2784 |
| CYP2D6 | 1080985 | 0.3000 | 16 | 0.0350 | 10, 13 | 0.2650 |
| ABCC2 | 2273697 | 0.3070 | 8 | 0.0430 | 19 | 0.2640 |
| CYP3A5 | 10264272 | 0.2420 | 11 | 0.0000 | 1, 2, 5-8, 18-21 | 0.2420 |
| CYP2A6 | 28399433 | 0.2790 | 18 | 0.0440 | 5  (the next is 1) | 0.2350 |
| CYP2C8 | 11572103 | 0.2350 | 10 | 0.0000 | 2, 3, 6, 14-15, 18-21 | 0.2350 |
| CYP2C9 | 1799853 | 0.2156 | 1 | 0.0000 | 10-13, 18-19, 21 | 0.2156 |
| SLCO1B1 | 4149056 | 0.1985 | 2 | 0.0000 | 10, 13 | 0.1985 |
| SLC22A2 | 316019 | 0.2470 | 13 | 0.0530 | 16 | 0.1940 |
| UGT1A1 | 4148323 | 0.1922 | 20 | 0.0000 | 1, 3-5, 8, 10-13, 16-17 | 0.1922 |
| CYP2C19 | 3758581 | 0.1832 | 1 | 0.0000 | 10, 13 | 0.1832 |
| SLC22A1 | 2282143 | 0.1742 | 18 | 0.0050 | 4 | 0.1692 |
| CYP2C8*3 | 10509681 | 0.1647 | 1 | 0.0000 | 10-13, 18-19, 21 | 0.1647 |
| CYP2C9 | 2256871 | 0.1460 | 11 | 0.0000 | 1-9, 14-16, 18-21 | 0.1460 |
| CYP2A6 | 28399454 | 0.1460 | 10 | 0.0000 | 1-9, 15-16, 18-19, 21 | 0.1460 |
| NAT2 | 1801279 | 0.1420 | 10 | 0.0000 | 1, 2, 5-9, 15-16, 18-19, 21 | 0.1420 |
| CYP2D6 | 28371725 | 0.1450 | 3 (followed by 1) | 0.0040 | 10 | 0.1410 |
| ABCB1 | 3213619 | 0.1470 | 13 | 0.0117 | 1 | 0.1353 |
| GSTP1 | 1138272 | 0.1353 | 1 | 0.0000 | 10, 12, 13, 18-20 | 0.1353 |
| TPMT | 1142345 | 0.1160 | 11 | 0.0000 | 1 | 0.1160 |
| CYP2B6 | 8192709 | 0.1284 | 1 | 0.0160 | 15 | 0.1124 |
| CYP2B6 | 28399499 | 0.0970 | 10 | 0.0000 | 1-9, 16, 18-21 | 0.0970 |
| SLC22A6 | 11568626 | 0.0840 | 10 | 0.0000 | 1-9, 14-15, 18-21 | 0.0840 |
| CYP1A1 | 1799814 | 0.0840 | 4 | 0.0000 | 6, 10-13, 18-21 | 0.0840 |
| CYP2C8 | 1058930 | 0.0810 | 2 | 0.0000 | 9-11, 13, 18-21 | 0.0810 |
| CYP2C19 | 4986893 | 0.0750 | 19 | 0.0000 | 1 -5, 10-11, 13-17 | 0.0750 |
| TPMT | 1800460 | 0.0650 | 16 | 0.0000 | 7-8, 10-13, 18-21 | 0.0650 |
| NAT1 | 4987076 | 0.0635 | 7 | 0.0000 | 1 -2, 10, 12-13 | 0.0635 |
| NAT1 | 4986988 | 0.0635 | 7 | 0.0000 | 2, 10, 12-13 | 0.0635 |
| NAT1 | 4986989 | 0.0635 | 7 | 0.0000 | 2, 10, 12-13 | 0.0635 |
| NAT1 | 4986990 | 0.0635 | 7 | 0.0000 | 2, 10, 12-13 | 0.0635 |
| SLC22A2 | 8177517 | 0.0630 | 12 | 0.0000 | 1-9, 15-21 | 0.0630 |
| SLC22A1 | 12208357 | 0.0629 | 5 | 0.0000 | 10-13, 18-19, 21 | 0.0629 |
| SLCO1B1 | 59502379 | 0.0582 | 12 | 0.0000 | 1-9, 14-15, 18-21 | 0.0582 |
| SLC22A1 | 34059508 | 0.0478 | 1 | 0.0000 | 6-13, 18-21 | 0.0478 |
| CYP2A6 | 1801272 | 0.0470 | 3 | 0.0000 | 8, 10-14, 18-21 | 0.0470 |
| CYP2D6 | 5030655 | 0.0450 | 2 | 0.0000 | 1, 6-7, 9-13, 15-21 | 0.0450 |
| TPMT | 56161402 | 0.0335 | 12 | 0.0000 | 1-9, 13, 15, 17-21 | 0.0335 |
| SLC22A1 | 34130495 | 0.0330 | 4 | 0.0000 | 7-13, 18-21 | 0.0330 |
| UGT1A9 | 35350960 | 0.0320 | 19 | 0.0000 | 1 -17 | 0.0320 |
| NAT1 | 5030839 | 0.0280 | 4 | 0.0000 | 1, 2, 5-9, 11-13, 17-21 | 0.0280 |
| CYP2C9 | 28371686 | 0.0266 | 12 | 0.0000 | 1-9, 14-16, 18-21 | 0.0266 |
| CYP2D6 | 5030865 | 0.0250 | 21 | 0.0000 | 1 -17 | 0.0250 |
| SLC22A2 | 8177516 | 0.0219 | 12 | 0.0000 | 1-3, 5-9, 11, 13-21 | 0.0219 |
| CYP2C9 | 9332131 | 0.0195 | 12 | 0.0000 | 1-9, 11, 13-21 | 0.0195 |
| ABCC2 | 56220353 | 0.0195 | 20 (21 also has MAF > 0) | 0.0000 | ALL OTHERS (except 21) | 0.0195 |
| NAT1 | 56172717 | 0.0140 | 4 | 0.0000 | 1, 3, 5-16, 18-21 | 0.0140 |
| CYP2D6 | 5030867 | 0.0120 | 6 | 0.0000 | 1 -5, 10-21 | 0.0120 |
| SLC22A1 | 55918055 | 0.0110 | 5 | 0.0000 | ALL OTHERS | 0.0110 |
| TPMT | 1800462 | 0.0110 | 5 | 0.0000 | 1 -2, 6-13, 15, 18-21 | 0.0110 |
| UGT1A9 | 34993780 | 0.0110 | 19 | 0.0000 | 1-5, 7-18, 21 | 0.0110 |
| CYP3A4 | 55785340 | 0.0100 | 2 | 0.0000 | ALL OTHERS | 0.0100 |
| SLC22A1 | 4646278 | 0.0100 | 8 | 0.0000 | 1 -3, 5-6, 10-21 | 0.0100 |
| SLC22A1 | 4646277 | 0.0100 | 18  (20 and 21 also have MAF > 0) | 0.0000 | ALL OTHERS except 20 and 21 | 0.0100 |
| SLC22A2 | 8177507 | 0.0090 | 10 | 0.0000 | ALL OTHERS | 0.0090 |
| CYP2C9 | 9332239 | 0.0090 | 3 (17 also have MAF > 0) | 0.0000 | ALL OTHERS except 17 | 0.0090 |
| CYP2C19 | 28399504 | 0.0080 | 15 | 0.0000 | 1-3, 5-14, 16, 18-19, 21 | 0.0080 |
| NAT1 | 56379106 | 0.0050 | 5 | 0.0000 | ALL OTHERS | 0.0050 |
| CYP2B6 | 12721655 | 0.0050 | 8 | 0.0000 | ALL OTHERS | 0.0050 |
| CYP1A1 | 72547509 | 0.0050 | 19 | 0.0000 | ALL OTHERS | 0.0050 |
| CYP2E1 | 72559710 | 0.0050 | 20, 21 | 0.0000 | ALL OTHERS | 0.0050 |
| SULT1A1 | 143283780 | 0.0048 | 12 | 0.0000 | ALL OTHERS | 0.0048 |
| DPYD | 1801267 | 0.0025 | 7 | 0.0000 | ALL OTHERS | 0.0025 |
| CYP2C19*8 | 41291556 | 0.0025 | 7 | 0.0000 | ALL OTHERS | 0.0025 |
| ABCC2 | 56296335 | 0.0025 | 20 | 0.0000 | ALL OTHERS | 0.0025 |
